# CRISPR/Cas-based genome editing for enhancing grain quality traits in rice (*Oryza sativa* L.)

**DOI:** 10.1080/15592324.2026.2713868

**Published:** 2026-08-13

**Authors:** Mahalakshmi Mani, S. Poonguzhali, K. Baghyalakshmi

**Affiliations:** a VIT School of Agricultural Innovations and Advanced Learning (VAIAL), Vellore Institute of Technology, Tamil Nadu, India; b ICAR-Central Institute for Cotton Research, Regional Station, Coimbatore, Tamil Nadu, India

**Keywords:** CRISPR/Cas, amylose content, grain size, aroma, chalkiness

## Abstract

Rice is one of the most important food crops and feeds more than half of the world’s population. Enhancing grain quality is currently a highly important issue since consumers are now more concerned with the taste, appearance, and nutritional value of the grain. The quality of grain in rice is complex and regulated by a multitude of genes that influence qualities such as amylase content, grain size and shape, chalkiness, aroma and nutrient content. The traditional forms of breeding, such as hybridization and marker-assisted selection, are slow and less effective since such characteristics are regulated by many genes and are influenced by environmental conditions. CRISPR/Cas genome editing has become a potent tool that enables scientists to directly and specifically edit grain quality-related genes. Important genes such as *Wx* (amylose), *GS3, GW8*, and *TGW3* (grain size), *Chalk5* (chalkiness), *BADH2* (aroma), and nutrient-related grain size genes such as *OsAAP6, OsAAP10*, and *OsVIT1/2*, have been successfully edited to enhance the quality of rice. Newer methods, such as base editing and prime editing, enable this process to become even more precise by modifying the specific bases of DNA without cutting the DNA. CRISPR has assisted in the improvement of rice by controlling the amylose content, reducing chalkiness, enhancing aroma, and improving nutritional quality. It is more accurate and quicker than traditional breeding, and it can enhance various traits simultaneously. Nonetheless, other challenges, such as off-target effects, regulatory concerns, and acceptance by the people, still have to be overcome. Combining CRISPR with artificial intelligence and genomic selection in the future will aid in creating superior versions of rice in shorter periods of time. Overall, CRISPR/Cas genome editing is a potential method to enhance the quality of rice and secure food security in the whole world.

## 1. Introduction

Rice (*Oryza sativa* L.) is a crop that has been grown for thousands of years and continues to be a staple of food systems all over the world, especially in Asia and parts of Africa. In addition to rice being a staple crop, rice plays a major role in livelihoods, trade, and cultural practices. Although earlier breeding programs mainly focused on increasing yield to satisfy food demand, more recent trends are characterized by a growing emphasis on the quality of grains due to changing consumer preferences and market demands.^1^ ^,^ ^2^ Rice grain quality involves a multi-dimensional facet of quality that involves physical appearance (grain size, shape, and translucency), cooking and eating properties (texture, stickiness, and gelatinization behavior), sensory attributes such as aroma, and nutritional components, such as protein and micronutrients.^3^ ^,^ ^4^These traits are polygenic and highly sensitive to environmental factors such as temperature and water availability that can greatly affect grain development and expression of quality.

The conventional breeding methods, such as hybrids and the use of markers, have been significant in enhancing the characteristics of rice. However, these methods are frequently constrained in their capacity to manipulate complex characteristics accurately, especially those that are controlled by multiple genes and are influenced by the interactions of the genotype and the environment.^5^ Furthermore, the lengthy process of introgressing desirable alleles into elite cultivars is time-consuming and may cause linkage drag, which decreases breeding efficiency. With the introduction of genome editing technologies, in particular, CRISPR/Cas systems, a transformative solution for crop improvement has been introduced. CRISPR/Cas allows targeted and efficient editing of specific genomic regions, and direct manipulation of genes linked to important agronomic and quality traits.^6^ ^,^ ^7^ Genome editing has helped rice to validate its functional candidates and to develop better varieties with desirable characteristics.

More recent innovations, such as base editing and prime editing, have further improved the accuracy of genome editing by allowing targeted nucleotide substitutions without causing a double-strand break.^8^ ^,^ ^9^ Also, multiplex genome editing techniques enable the concurrent editing of multiple genes, which opens up opportunities to enhance complex traits, which are regulated by networks of genes rather than individual loci.^10^ In spite of these developments, several challenges persist, such as the concerns over off-target effects, the effectiveness of plant transformation and regeneration, and different regulatory frameworks across nations.^11^ The way genome-edited crops are taken up also depends on how they are perceived and accepted by people. In this regard, the application of CRISPR/Cas-based genome editing for enhancing the quality of rice grains is reviewed. This research seeks to address the genetic foundations of important quality characteristics, the innovations in genome editing tools, and their applications, along with the prevailing challenges and future prospects in precision rice breeding.

## 2. Genetic basis of grain quality traits in rice

A complicated system of genes that regulate the biosynthesis of starch, grain morphology, aroma, chalkiness, as well as the nutritional composition, governs the quality of grains in rice. These characteristics are polygenic, and are mediated by coordinated biochemical and physiological mechanisms.^3^ ^,^ ^4^

### 2.1. Amylose level and starch biosynthesis

The Waxy (*Wx*) gene is the one that encodes granule-bound starch synthase I (GBSSI), which is involved in amylose synthesis in the rice endosperm.^12^ The amylose content is determined by variation in *Wx* expression, classifying rice into glutinous, low, intermediate, and high-amylose types.^12^ Soluble starch synthases (SS), starch branching enzymes (SBE), and debranching enzymes (DBE) are other essential enzymes involved in the biosynthesis of amylopectin and starch granules.^13^ The regulatory proteins, including FLOURY ENDOSPERM 6 (*FLO6*), help in the organization of starch granules.^14^

### 2.2. Grain size and shape

The grain size is controlled by genes that regulate cell proliferation and expansion when developing grains. *GS3* is a negative regulator of grain length, and *GW2* encodes an E3 ubiquitin ligase, which negatively regulates grain width by controlling cell division.^15^ ^,^ ^16^ The *GW5/qSW5* locus is a locus that affects grain width by modulating cell proliferation.^17^ Grain size is increased by positive regulators such as *GS5* and *GL7/GW7*, which promote cell growth.^18^ ^,^ ^19^ Other genes, including *TGW6* and *DEP1*, which have an effect on grain weight and panicle architecture.^20^ ^,^ ^21^

### 2.3. Chalkiness

Chalkiness is caused by the starch granules in the endosperm being loosely packed, resulting in an opaque appearance of the grain. The *Chalk5* gene contains a vacuolar H+ -pyrophosphatase, which regulates the development of endosperm and the deposition of starch.^22^ The *FLO2* gene is the gene encoding a tetratricopeptide repeat (TPR), a protein that regulates the starch biosynthesis and the storage protein accumulation.^23^ Other genes that are involved in starch metabolism include *SSIIIa*, *PUL*, and *UGPase1.* ^24^

### 2.4. Aroma (fragrance)

The buildup of 2-acetyl-1-pyrroline (2AP) is the primary determinant of rice fragrance. *BADH2* gene is the gene that encodes betaine aldehyde dehydrogenase, which catalyzes the conversion of *γ*-amino butyraldehyde into *γ*-amine butyric acid (GABA).^25^ Mutation of *BADH2* leads to the accumulation of 2AP to form the typical aroma of aromatic varieties of rice.^1^ ^,^ ^25^

### 2.5. Nutritional quality (iron, zinc, and protein)

**Figure 1.**
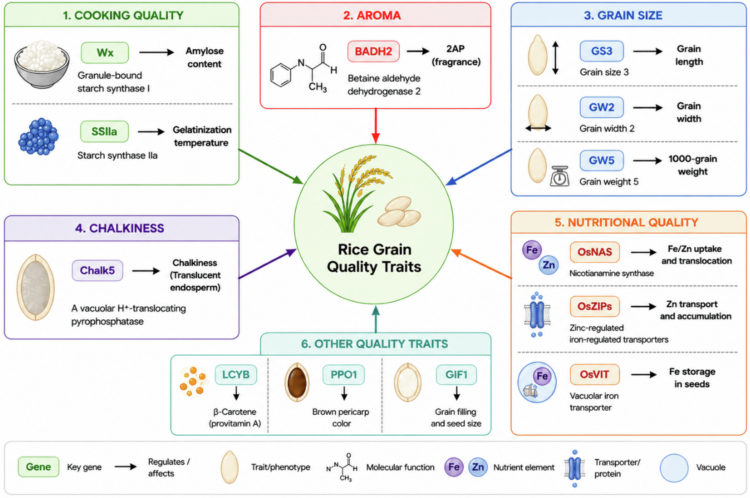
This integrated view highlights the complex genetic architecture governing grain quality traits and provides a foundation for targeted genome-editing strategies.

The genes that regulate the accumulation of micronutrients in rice grains control the uptake, transport, and storage of the micronutrients in rice grains. The *OsNAS* genes enable the process of metal chelation and transportation (Figure 1).^26^ The movement and storage of iron and zinc in developing grains are controlled by transporters, including *OsZIP*, *OsYSL*, and *OsVIT* ^27-29^. Storage of protein genes, such as glutelins and prolamins, determines the protein content in grains, nutritional quality, and digestibility.^30^

## 3. Overview of CRISPR/Cas genome editing systems

CRISPR/Cas systems offer flexible platforms for editing the genomes of plants. The canonical CRISPR/Cas9 system involves a single-guide RNA (sgRNA) to direct the Cas9 nuclease toward a complementary DNA target adjacent to a protospacer adjacent motif (PAM, typically NGG). Cas9 forms a double-strand break (DSB), which is repaired by non-homologous end joining (NHEJ) or homology-directed repair (HDR). NHEJ results in small deletions/insertions (indels) that cause gene knockouts; HDR can insert supplied donor DNA to make precise edits (although HDR is inefficient in plants).

In addition to Cas9, there are other CRISPR nucleases with distinct characteristics. Cas12a (Cpf1) recognizes T-rich PAMs (TTTV) and makes staggered cuts with sticky ends. It is possible to process its own crRNA array, allowing easy multiplex editing.^31^ Cpf1-based editors can be used to target genomic regions that would be inaccessible to Cas9. Newer versions of CRISPR have the ability to be precise at the base level. Base editors merge a catalytically impaired Cas (nCas9) with a cytidine or adenosine deaminase to produce C to T (cytidine base editor, CBE) or A to G (adenine base editor, ABE) at specific sites without DSBs.^31^ Rice has been used to introduce point mutations that affect traits (e.g., herbicide resistance, yield QTLs). They can be used to mimic natural SNPs. Prime editing involves a Cas9 nickase fused with reverse transcriptase and a prime editing guide RNA (pegRNA) to search-and-replace DNA, allowing insertions, deletions, or replacements without DSBs (although prime editing is still in its infancy in plants). The benefits of CRISPR are programmability, high efficiency, multiplexing, and, in many cases, lower cost than older techniques (ZFNs, TALENs).^32^ For example, four yield genes (*Gn1a, DEP1, GS3*, and *IPA1*) could be multiplex edited in a single transformation.^33^ A comparative overview of the major CRISPR/Cas genome editing systems, their editing mechanisms, advantages, and applications in rice is presented in Table 1.

**Table 1.** Advanced overview of CRISPR/Cas systems for rice genome editing.

CRISPR system	Enzyme/Component	PAM requirement	Editing mechanism	Type of modification	Advantages	Applications in Rice	Key References
**CRISPR/Cas9**	SpCas9 endonuclease + sgRNA	NGG	Introduces double-strand breaks (DSBs) repaired via NHEJ/HDR	Gene knockout (InDels), gene insertion/replacement	High efficiency, widely validated, easy design	Editing *Wx* (amylose), *BADH2* (aroma), *GS3, GW2* (grain size)	[10,12,34,35]
**CRISPR/Cas12a (Cpf1)**	Cas12a nuclease + crRNA	TTTV	Creates staggered DSBs with sticky ends	Gene disruption, targeted insertions	Higher specificity, multiplex editing, smaller RNA	Multi-gene editing and trait stacking in rice	[32,36]
**Base Editing (CBE/ABE)**	Cas9 nickase fused with deaminase	NGG (or variants)	Direct base conversion without DSBs	C→T (CBE), A→G (ABE) substitutions	High precision, low off-target, no DSB	Fine-tuning *Wx* alleles, improving grain quality traits	[8,37]
**Prime editing**	Cas9 nickase + reverse transcriptase + pegRNA	Flexible	Reverse transcription-mediated precise editing	Insertions, deletions, base substitutions	Highly precise, versatile, no donor DNA require	Emerging tool for precise editing of grain quality genes	[9]
**Multiplex CRISPR**	Multiple sgRNAs with Cas9/Cas12a	Depends on system	Simultaneous targeting of multiple loci	Multi-gene editing	Enables pyramiding of traits	Editing multiple quality genes (*Wx, BADH2, GS3*) simultaneously	[10,38]
**CRISPR/dCas9 (Gene Regulation)**	Dead Cas9 (dCas9) fused with activators/repressors	NGG	No DNA cutting; transcriptional regulation	Gene activation/repression (CRISPRa/CRISPRi)	Reversible, non-mutagenic	Regulation of grain quality pathway	[39]

CRISPR/Cas genome editing tools represent a wide range of tools that allow specific and precise genetic editing in rice, which allows enhancing complex grain quality characteristics. The most common of these is CRISPR/Cas9, which is a simple, effective, and versatile tool to produce gene knockouts and targeted changes using the DSB-mediated repair pathways.^10^ ^,^ ^35^ CRISPR/Cas12a (Cpf1), in its turn, has several advantages, such as staggered cleavage of DNA, specific PAM recognition (TTTV), and multiplexing; thus, it can be used to simultaneously edit a number of genes that regulate grain quality.^36^ Newer developments like base editing and prime editing have further increased the accuracy of genome editing by allowing specific nucleotide replacements and sequence alteration without the formation of DSBs, thus reducing off-target effects and genomic instability.^9^ ^,^ ^38^ In addition, multiplex CRISPR approaches can be employed to modify several loci simultaneously, and this is particularly useful in improving polygenic traits such as amylose content, grain size, and aroma in rice.^38^ Genome editing can now be extended to transcriptional regulation, using new technologies like CRISPR/dCas9-based systems, which do not require the modification of the DNA sequences.^39^ Taken together, these CRISPR/Cas systems offer a complete breeding toolkit to precisely target the genetic modification of rice to expedite the creation of high-quality rice varieties with enhanced nutritional, cooking, and marketing characteristics.

## 4. CRISPR/Cas workflow in rice

**Figure 2.**
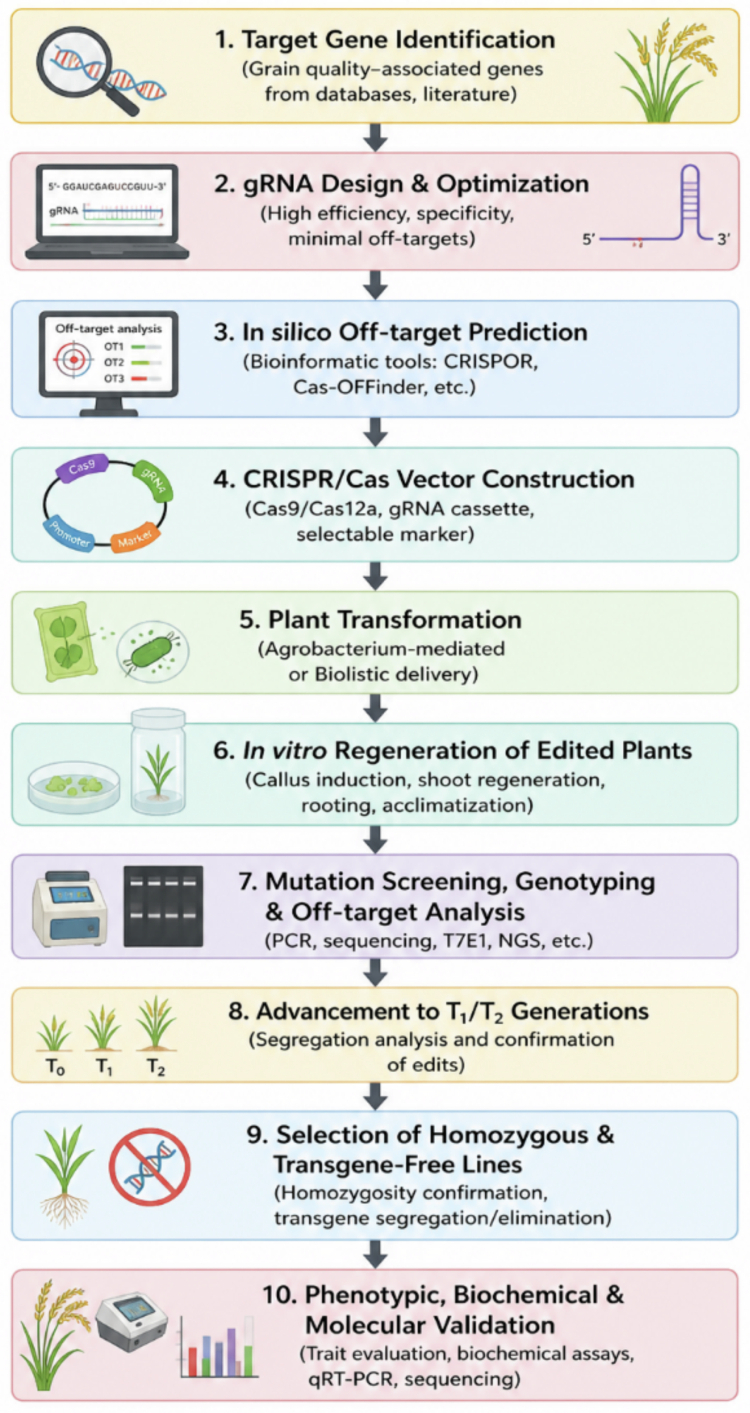
General workflow of CRISPR/Cas-mediated genome editing in rice for grain quality improvement, including target identification, editing, screening, and validation steps.

An average CRISPR editing workflow in rice consists of the following steps (Figure 2).

### 4.1. Target selection

Find candidate genes/QTLs that influence the desirable trait (e.g., *Wx* to amylose, *BADH2* to aroma). Gene activity was confirmed through either the literature or initial experiments.

### 4.2. gRNA design

Guide RNAs using software (CRISPR-*P*, CHOPCHOP, E-CRISP) to design guide RNAs with a high on-target score and low off-targets. Screen the genome with potential off-target sites of check rice.

### 4.3. Vector construction

The sgRNA sequence(s) were synthesized and cloned into a plant binary vector containing a rice codon-optimized Cas9 (or Cas12a) driven by a strong promoter (e.g., *OsU6* to promote sgRNA). In the case of multiplex editing, several sgRNA cassettes can be constructed.

### 4.4. Plant transformation

The construct can be transferred into rice callus using *Agrobacterium tumefaciens*. Transgenic regenerated plants were selected. The variety of tissue culture conditions and selectable markers (hygromycin, etc.) is dependent on the variety.

### 4.5. Screening mutants

In T_0_ plants, PCR amplifies target loci and sequences to determine mutations. Indels can be identified using assays (e.g., T7E1, PAGE, and Sanger sequencing). Identifying homozygous/biallelic mutants. To eliminate plants with T-DNA, we wish to obtain transgene-free lines.

### 4.6. Validation

Grow to maturity-edited lines. Phenotypes (measures of grain quality) and genotypes were determined. Sequencing was used to confirm on-target edits and check the top potential off-target sites. Confirm changes in traits by performing biochemical assays (e.g., starch content by SEC, 2AP quantification by GC–MS). In one study, CRISPR/Cas9 knocked out two parental lines of *Wx* and *BADH2* simultaneously, resulting in two parental lines of double mutants that produced true amylose 0.22%–1.63% (vs −13% WT) and had 2AP aroma of −150 µg/kg.^12^

## 5. Trait-wise CRISPR applications in rice grain quality

### 5.1. Amylose content modification

Promoter editing with CRISPR/Cas9 generated 14 new *Wx* alleles that produced amylose contents of 5.8%–9.6%. In a different study, Zhang et al. found that *Wx* knockout resulted in glutinous rice with a low amylose content (below 2%), with no significant effects on agronomic performance. The results show that CRISPR-based strategies are effective in fine-tuning the composition of starch.^12^ Representative examples of CRISPR/Cas-mediated editing for major rice grain quality traits, together with their quantitative outcomes, are summarized in Table 2.

### 5.2. Grain size improvement

Huang et al. created *GS3* knockout lines using CRISPR/Cas9, and the results showed that the grain length increased by 7.9%, and the thousand-grain weight increased by 6.7%.^40^ *GS3* and *GW5* were edited by Zeb et al., creating longer and wider grains with higher yields.^41^ Moreover, Ludwig et al. showed multiplex genome editing in *GS3, GW2,* and *Gn1a,* which resulted in a significant increase in grain number per panicle.^33^

### 5.3. Reduction of chalkiness

Grain chalkiness is an undesirable quality trait that adversely affects grain appearance, milling quality, and consumer acceptance. Multiplex CRISPR/Cas-mediated editing of *Chalk5*, *GW2*, and *TGW3* significantly reduced grain chalkiness and improved grain transparency, demonstrating that simultaneous editing of multiple grain quality-related genes is an effective strategy for improving rice grain appearance.^42^

### 5.4. Aroma enhancement

Ashokkumar et al. created targeted mutations in *BADH2* under the CRISPR/Cas9 system, leading to the generation of fragrant rice lines with improved accumulation of 2AP.^43^ Zhang et al. further showed that *BADH2* knockout in hybrid rice parental lines produced about 150 µg/kg of 2AP, which demonstrated that there were no adverse effects on the yield.^12^

### 5.5. Nutritional enhancement (biofortification)

Achary and Reddy reported that a *GW2* knockout in the CRISPR-mediated knockout showed increased grain size and improved iron and zinc content due to the thickening of the aleurone layer.^34^ Ludwig et al. employed the promoter editing of *OsNAS2*, which led to higher levels of zinc accumulation and yield.^33^ These papers show the promise of CRISPR-based solutions to concurrently enhance nutritional quality and productivity.

**Table 2.** Quantitative outcomes of CRISPR/Cas-mediated editing for rice grain quality improvement.

Target gene	Trait improved	Editing method	Quantitative outcome	Reference
*Wx*	Amylose content	CRISPR/Cas9 knockout	Amylose reduced from ~13% to <2%	[12]
*Wx* promoter	Cooking quality	Promoter editing	Amylose fine-tuned to 5.8–9.6%	[44]
*GS3*	Grain length	CRISPR/Cas9 knockout	Grain length increased by 7.9%; TGW increased by 6.7%	[40]
*BADH2*	Aroma	CRISPR/Cas9 knockout	2AP increased to ~150 µg/kg	[12,43]
*Chalk5*	Chalkiness	Multiplex editing	Reduced grain chalkiness and improved transparency	[42]
*OsNAS2*	Zinc biofortification	Promoter editing	Increased Zn accumulation and yield	[33]

## 6. Applications and molecular mechanisms of CRISPR/Cas-mediated grain quality improvement in rice

**Figure 3.**
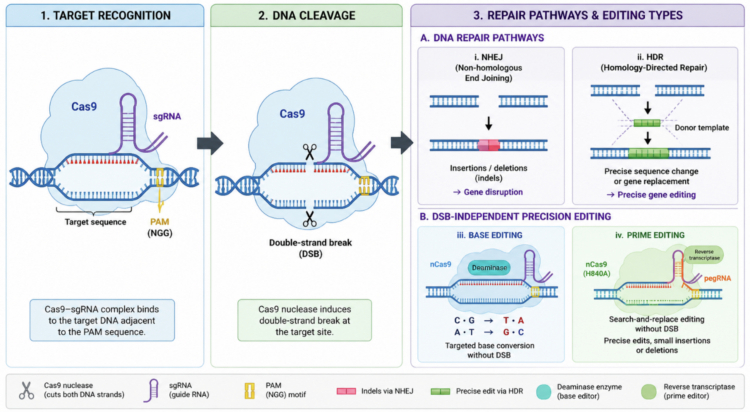
The molecular mechanism of CRISPR/Cas-mediated genome editing, including target recognition, DNA cleavage, and repair pathways.

CRISPR/Cas technology has emerged as an efficient and precise method to enhance the rice grain quality traits. Carbohydrate metabolism, secondary metabolite biosynthesis, protein accumulation, and micronutrient transport are interconnected molecular pathways that regulate grain quality traits, including cooking quality, texture, aroma, grain appearance, and nutrition. CRISPR/Cas systems can target genes that affect these pathways, allowing for more precise manipulation of grain quality attributes and more rapid improvement of rice programs (Figure 3). CRISPR/Cas is a system of prokaryotic adaptive immunity that is highly specific in terms of RNA/DNA recognition and cleavage of DNA. The system is composed primarily of a Cas nuclease (e.g., Cas9 and Cas12a) and a single-guide RNA (sgRNA) that guides the nuclease to a complementary genomic sequence next to a protospacer adjacent motif (PAM). The PAM sequence for Streptococcus pyogenes Cas9 (SpCas9) is often 5′-NGG-3′.^45^ ^,^ ^46^ After target recognition, Cas9 creates a PAM sequence-specific DSB approximately 3 base pairs upstream of the PAM sequence, which triggers endogenous DNA repair pathways.

There are two major mechanisms of DNA repair that are necessary for CRISPR/Cas-mediated editing, namely, NHEJ and HDR. NHEJ is the most common repair process in plants and frequently causes insertions or deletions (indels) that alter the function of the gene by frameshift mutations or premature stop codons. Thus, NHEJ is widely adopted in rice gene knockout strategies for rice improvement. In contrast, HDR allows precise modification of a sequence by using a donor template of homologous DNA, but the efficiency of HDR in rice is still low.

Recent developments such as base editing and prime editing, have further enhanced the precision of editing. In base editing systems, a catalytically compromised Cas protein is fused to a deaminase enzyme to allow direct nucleotide substitutions without generating DSBs, eliminating the need for the DSBs that are often required by other base editing technologies.^47^ ^,^ ^48^ Prime editing is another form of editing that allows for targeted insertions, deletions, and base substitutions by combining a reverse transcriptase with Cas9 nickase and a pegRNA.^49^ Moreover, multiplex genome editing, which allows multiple genes to be modified simultaneously with multiple sgRNAs, can be used to coordinate the modification of complex polygenetic traits, including grain texture, chalkiness, aroma, and nutritional quality.^38^ ^,^ ^50^ ^,^ ^51^

The starch biosynthesis pathway is one of the most critical pathways that affects the cooking and eating quality of rice. Amylose and amylopectin accumulation in the endosperm are regulated by enzymes including GBSSI (encoded by *Wx*), SS, SBE, and starch debranching enzymes (DBE). Direct alteration of the Wx gene by CRISPR/Cas changes the amylose content, which in turn affects grain texture, viscosity, and gelatinization properties. Similarly, changes in *SSIIa* and *SBEIIb* lead to changes in amylopectin structure and branching, which changes the gelatinization temperature and cooking properties.^52^ These changes allow the breeding of new varieties of rice with improved cooking characteristics, such as soft, sticky, or fluffy texture.

Another pathway that is important for rice grain quality is aroma biosynthesis. Betaine aldehyde dehydrogenase (*BADH2*), which encodes a fragrance in rice, is the major gene controlling the fragrance. *BADH2* loss-of-function mutations block normal metabolic pathways and lead to the buildup of 2-acetyl-1-pyrroline (2-AP), the key aromatic compound that gives fragrance to rice. CRISPR/Cas9 technology has been employed to disrupt the *BADH2* gene successfully in order to introduce aroma in non-aromatic elite rice varieties without compromising on agronomic traits.^43^ Here, the direct connection between genome editing and metabolic pathway modification has been shown for sensory quality traits.

Grain size and grain shape are significant factors in market value, milling quality, and consumer preference. These genes are regulated by complicated signaling pathways that control cell division, cell expansion, and hormone action that occur during grain development. The genes *GS3, GW2*, and *GW5* control grain length and grain width through ubiquitin-mediated protein degradation pathways and G-protein signaling pathways. These genes are edited to affect grain cell growth and proliferation, thus affecting the grain shape, grain weight, and milling quality of the grains.^53^ Likewise, genes involved in endosperm development (e.g., *Chalk5*) and starch granule packing (e.g., transparency) affect grain chalkiness and transparency.

Protein biosynthesis and storage pathways are also important factors for grain quality. The nutritional value, digestibility, and cooking properties of the storage proteins glutelins, prolamins, and albumins are affected. The nutritive value of rice-based diets can be improved by CRISPR/Cas-based editing of storage protein genes, which can also modify the composition of the protein, make it more digestible and less allergenic.^54^ The accumulation of micronutrients in rice grains is regulated by pathways involving metal uptake, transport, and storage. Iron and zinc mobilization and deposition in the endosperm are controlled by important genes such as *OsNAS* (nicotianamine synthase), *OsZIP* (zinc transporter), *OsYSL* (yellow stripe-like transporter), and *OsVIT* (vacuolar iron transporter). The incorporation of these genes into the genome by the CRISPR/Cas system may enhance nutrient uptake, translocation, and grain loading efficiency, thus improving micronutrient biofortification.^55^ Additionally, lowering anti-nutritional factors such as phytic acid, may enhance mineral bioavailability even more.

Transcription factors, including the BZIP, NAC, and MYB families, also play a role in grain quality traits via regulatory networks. These transcription factors control the expression of downstream genes in response to developmental and environmental signals. CRISPR/Cas technology can be used to edit regulatory elements such as promoters and enhancers to precisely modulate gene expression without changing their coding sequence. These methods are especially beneficial for the fine-tuning of complex traits where full knockout of the gene in question might have unwanted side effects. The efficiency of CRISPR/Cas-mediated editing is dependent on several factors, such as the sgRNA design, GC content, chromatin accessibility, off-target potential, and type of Cas protein used. Recent studies have also demonstrated that the epigenetic context influences editing efficiency, which may be due to the inaccessibility of the DNA target to Cas in regions of tightly packed chromatin.^56^ In addition, engineered Cas variants with relaxed PAM requirements (e.g., SpCas9-NG and xCas9) have extended the targetable window of the rice genome and increased the targeting flexibility of quality-related genes.

In summary, the genome editing technology of CRISPR/Cas is a very promising tool for the engineering of molecular pathways involved in rice grain quality. There are great opportunities for the development of rice varieties with better cooking quality, better aroma, better grain appearance, and better nutritional value because of the integration of pathway-level knowledge with advanced genome editing technologies. The utilization of multi-omics approach and systems biology tools in the future will further enhance the prediction and engineering of complex grain quality traits in rice.

Rice grain quality is controlled by complex molecular pathways, not by individual genes. The starch biosynthesis pathway is the main mechanism responsible for the control of cooking quality, where the actions of *Wx* (GBSSI), *SSIIa*, SBE, and DBE regulate the synthesis of amylose and amylopectin, and affect the gelatinization temperature, texture, and eating quality. The *BADH2* pathway is the most important pathway for aroma in rice; loss-of-function mutations cause the buildup of the key aroma compound 2-acetyl-1-pyrroline (2AP) in fragrant rice. The genes that control grain appearance include *GS3, GW2, GW5* and *Chalk5* affecting cell proliferation, grain morphology, endosperm development and starch granule organization. Metal uptake and transport pathways (*OsNAS, OsZIP, OsYSL,* and *OsVIT*) control metal uptake, translocation, and storage in developing grains, and are fundamental to nutritional quality. CRISPR/Cas pathway editing technology allows for the simultaneous enhancement of several grain quality traits by precise modification of key grain quality regulatory genes.^1^ ^,^ ^6^ ^,^ ^8^ ^,^ ^33^

## 7. CRISPR/Cas systems used for rice improvement

The emergence of CRISPR-based genome editing technologies has tremendously increased the toolbox that can be used to specifically improve the genetic aspects of rice. The various CRISPR/Cas systems differ in their structure, PAM needs, editing efficiency, and application in specific breeding goals. These systems have been tactically utilized to alter the qualities of grains, which include starch composition, aroma, nutritional value, and grain morphology.

### 7.1. CRISPR/Cas9 system

The CRISPR/Cas9 system is the most commonly used genome editing tool in rice because of its simplicity, high efficiency, and reliability. Cas9 is derived from *Streptococcus pyogenes* and recognizes an NGG PAM sequence and creates DSBs at target loci. CRISPR/Cas9 has been significantly applied in gene knockout studies of rice, especially gene knockouts of genes related to grain quality. As an example, the process of editing the *BADH2* gene leads to an increase in aroma, and mutations in *Wx* determine the content and cooking properties of amylose.^43^ ^,^ ^53^ Although it is efficient, some of the limitations are its dependency on PAM and its possible off-target effects.

### 7.2. CRISPR/Cas12a (Cpf1) system

CRISPR/Cas12a (previously Cpf1) is a different system that has its own benefits over Cas9. It identifies a TTTV and is therefore appropriate for probing AT-rich regions within the rice genome. Cas12a produces staggered DNA cleavages with sticky endings, which can potentially permit more accurate insertions. Also, it needs a short CRISPR RNA (crRNA), making it easier to design constructs. Research has shown that Cas12a can be successfully used in rice to target mutagenesis and multiplex editing, especially with regard to the enhancement of the grain characteristics and stress resistance.^36^

### 7.3. Base editing technologies

Base editing is one of the significant improvements that permit the accurate replacement of the nucleotide without the creation of DSBs. Cytosine base editors (CBEs) are able to convert C•G to T•A, whereas ABEs can convert A•T to G•C. Base editing has been effectively used in rice to alter alleles of various genes that regulate grain quality characteristics, including starch biosynthesis genes and loci that influence nutrient properties.^57^ This method minimizes accidental mutations and enhances editing specificity, which is especially effective in fine-tuning gene function.

### 7.4. Prime editing

Prime editing is a next-generation technology of genome editing that can precisely insert, delete, and replace bases without the need for DSBs or donor templates. It uses a Cas9 nick of reverse transcriptase and a pegRNA. Though still in its early stages of implementation in plant systems, prime editing has already demonstrated encouraging outcomes in rice to perform specific gene editing. This technology has considerable potential for correcting allelic variations that influence grain quality traits with high accuracy.^49^

### 7.5. Multiplex genome editing

Multiplex genome editing involves the concurrent targeting of multiple genes with multiple single-guide guide RNAs (sgRNAs) in a single construct. This approach is especially useful for enhancing the complex characteristics, including the grain quality, that are controlled by numerous genes and routes. Multiplex editing has been applied in rice to simultaneously edit genes that determine grain size, yield, and quality, accelerating breeding programs and trait pyramiding.^38^ ^,^ ^50^

Many different studies have shown that multiplex genome editing is effective in rice for multiple crop improvement of agronomic and grain quality traits. Ma et al. developed a polycistronic tRNA–gRNA (PTG)-based CRISPR/Cas9 system that allows simultaneous editing of multiple target genes in rice plants by targeting them at the same time.^10^ In recent years, multiplexing of the CRISPR/Cas9 system has been used to edit multiple genes at the same time, including the grain quality genes *Wx, GS3, GW2*, and *GW5*, which led to simultaneous increases in the amylose content, grain size, and grain appearance. These multiplex editing techniques allow for pyramiding of desirable alleles in a single generation, thus saving breeding time and enhancing breeding efficiency for complex quantitative traits.^10^ ^,^ ^50^ ^,^ ^53^

### 7.6. CRISPR Interference (CRISPRi) and Activation (CRISPRa)

Cas proteins (dCas9) that are catalytically inactive have been engineered to regulate genes instead of editing the genome. CRISPR interference (CRISPRi) suppresses the expression of genes, whereas CRISPR activation (CRISPRa) activates transcription. These systems can be useful in regulating the level of gene expression without changing the DNA sequence. CRISPRi/a can be used to regulate rice genes involved in grain filling, starch metabolism, and nutrient transport, providing a reversible and tunable way to improve traits.

Several studies in plants have shown the potential of CRISPRi and CRISPRa. Lowder et al. developed dCas9-mediated transcription repression (CRISPRi) and activation (CRISPRa) systems to allow programmable manipulation of endogenous genes in plants, which would allow for functional genomics and crop improvement. Although direct applications of CRISPRi and CRISPRa in rice grain quality improvement are still limited, these technologies offer significant potential for reversible regulation of key genes, including *Wx*, *SSIIa*, *OsNAS2*, and *OsYSL2*, to improve cooking quality and nutritional traits without permanent genome modification.^39^

### 7.7. Ribonucleoprotein (RNP)-based delivery systems

**Figure 4.**
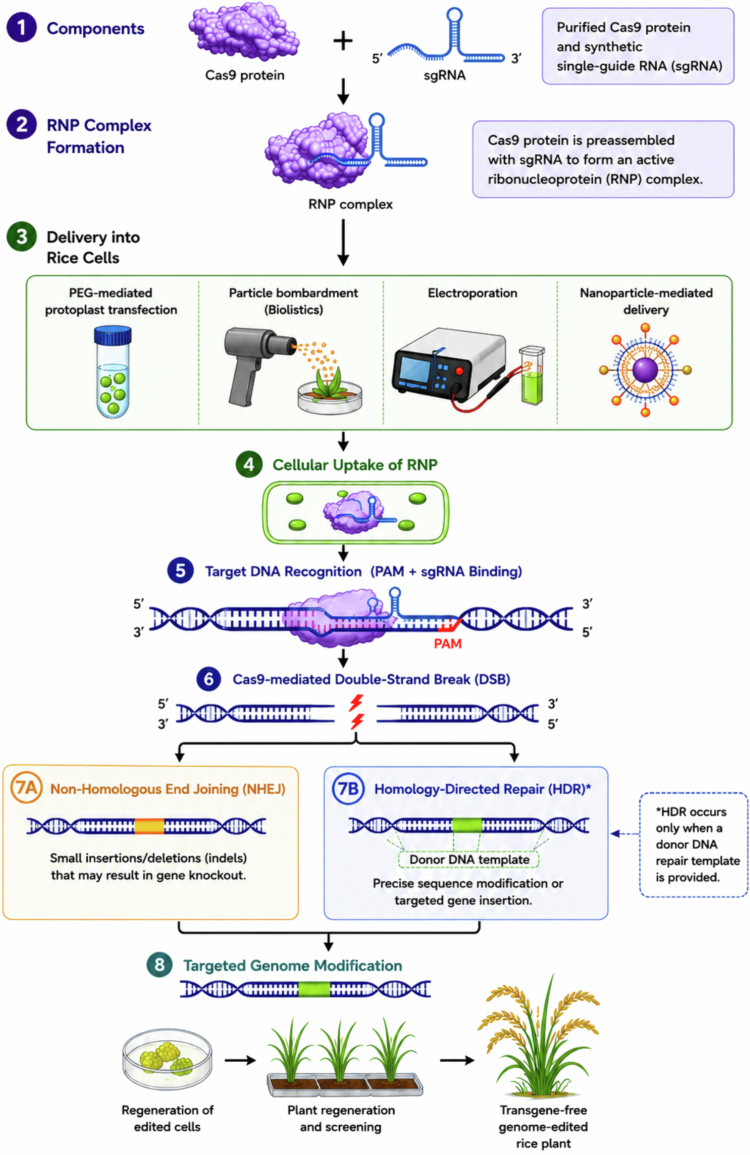
Workflow of ribonucleoprotein (RNP)-based CRISPR/Cas genome editing in rice (Created with BioRender).

The RNP-based genome editing system involves direct delivery of a preassembled complex of Cas9 protein and synthetic single-guide RNA (sgRNA) into rice cells without the introduction of foreign DNA. The RNP complex can be introduced into cells by polyethylene glycol (PEG)-mediated protoplast transfection, particle bombardment (biolistics), electroporation, or nanoparticles. After internalization, the Cas9-sgRNA complex binds to the target DNA near the protospacer adjacent motif (PAM) and cuts the DNA with DSB that can be repaired by both NHEJ and HDR. Transient activity of the RNP complex reduces off-target effects and avoids stable genomic integration, allowing efficient generation of transgenic rice plants that are free of transgenes. Figure 4 shows the genome editing procedure of RNP-mediated CRISPR/Cas in rice.

**Table 3.** Key gene targets for rice grain quality improvement using genome editing.

Trait	Gene/Protein target	Chromosome	Function	Editing strategy	Outcome	Reference
Amylose content	*Wx*	Chr 6	Encodes GBSSI for amylose synthesis	Knockout/promoter editing	Reduced amylose (<2%) or fine-tuned levels	[12,44]
Gelatinization temperature	*SSIIa*	Chr 6	Controls amylopectin structure	Base editing	Altered cooking quality	[52]
Aroma	*BADH2*	Chr 8	Regulates 2AP metabolism	Knockout	Increased fragrance	[43]
Grain length	*GS3*	Chr 3	Negative regulator of grain length	Knockout	Increased grain length	[40]
Grain width	*GW2*	Chr 2	Controls cell division	Knockout	Increased grain width and weight	[34]
	*GW5*	Chr 5	Regulates cell proliferation	Knockout	Increased grain width	[41]
Chalkiness	*Chalk5*	Chr 5	Affects starch deposition	Knockout	Reduced chalkiness	[42]
Iron and Zinc	*OsNAS2*	Chr 3	Metal chelation	Promoter editing	Increased Zn accumulation	[33]
Iron transport	*OsYSL2*	Chr 4	Metal transport	Gene regulation/editing	Improved Fe/Zn loading	[55]
Zinc transport	*OsZIP*	Multiple chromosomes	Zn uptake and transport	Editing	Enhanced Zn content	[28]
Protein content	Glutelins	Multiple chromosomes	Storage proteins	Knockout/editing	Modified protein composition	[54]

### 7.8. Designed and high-fidelity Cas variants

To increase the flexibility of targeting and minimize off-target effects, a series of engineered Cas variants have been developed, including SpCas9-NG, xCas9, and high-fidelity Cas9 (HiFi Cas9). The variants are more specific, and they identify wider PAM sequences. The use of these systems in rice increases the number of targetable genomic regions, allowing specific genes related to grain quality and nutritional enhancement to be precisely targeted.^58^

In general, the variety of CRISPR/Cas systems offers a toolkit for the complete improvement of rice. Although CRISPR/Cas9 continues to be the workhorse of genome editing, more recent technologies are beginning to be used more frequently to enhance precision and efficiency: base editing, prime editing, and multiplex approaches. The combination of these systems will provide an opportunity to target manipulation of complex grain quality attributes to open the way to next-generation rice varieties with superior nutritional and consumer-preferred characteristics.

## 8. Gene targets for grain quality improvement in rice

Genome editing methods have been employed to target a number of genes linked to traits of rice grain quality. These genes are involved in the regulation of important processes such as starch biosynthesis, determination of grain size, formation of aroma, chalkiness, as well as accumulation of micronutrients. The recent developments in genome editing technologies have made it possible to target these areas precisely to enhance the quality of the grain. In addition to trait improvement, genome editing has also been widely used for the functional validation of grain quality-related genes. For example, CRISPR/Cas9-mediated knockout of *OsCG5* increased grain chalkiness under heat stress, confirming its positive role in maintaining grain quality under elevated temperature conditions.^59^ Table 3 summarizes the major targets of gene editing, their functions, and the outcomes of editing.

Overview of target genes suggests that the activities of genome editing in rice are more or less concentrated on major regulators such as *Wx, GS3,* and *BADH2,* which have a significant and predictable phenotypic impact. The majority of studies use knockout strategies, as they reflect the predominance of non-homologous end joining-based strategies in plants. But new methods are coming along, such as base editing and promoter editing, which allow more fine-tuned and precise trait modification. Although these improvements have been made, at the same time, it has been difficult to simultaneously improve several grain quality characteristics because of multifaceted genetic interactions and possible pleiotropic effects. The next step of studying should be on multiplex editing and pathway-level engineering to ensure extensive grain quality improvement.

## 9. Recent CRISPR/Cas studies in rice grain quality improvement

The last few years have been associated with a high rate of application of CRISPR/Cas technologies to enhance grain quality traits in rice. These developments point to the emerging shift from simple gene knockouts to precise, multiplex, and allele-specific editing strategies, allowing improvement of multiple quality attributes, such as cooking quality, aroma, grain appearance, and nutritional value. The Waxy (*Wx*) gene, which regulates the synthesis of amylose, is one of the most studied targets. *Wx* editing via CRISPR/Cas9 has been used to produce rice lines with altered amylose levels, leading to an improved texture and eating quality. Promoter editing and base editing techniques have enabled the fine-tuning (instead of complete knockout) of *Wx*, with the desired intermediate amylase content preferred by consumers.^44^ ^,^ ^52^ Likewise, the editing of *SSIIa* has been employed to modify the gelatinization temperature, which additionally enhances the quality of cooking.

The *BADH2* gene continues to be a primary target of aroma improvement. Several studies have reported that CRISPR/Cas9-mediated knockout of *BADH2* increased the accumulation of the key aroma compound, 2-acetyl-1-pyrroline (2-AP) in fragrant rice. Notably, recent studies have been focused on the introduction of fragrance in high-yielding, non-aromatic cultivars without any impact on the agronomic performance, which indicates the practical utility of genome editing in breeding programs.^43^ ^,^ ^60^ CRISPR-based methods have also been widely used to improve grain size and appearance. The *GS3, GW2, GW5,* and *TGW6* genes are targeted to modify the grain length, width, and weight. The multiplex genome editing protocols have made it possible to modify several grain size genes at a time, leading to an optimized grain morphology and an enhanced ratio of yield-quality.^40^ ^,^ ^61^ Furthermore, the selective editing of *Chalk5* has been demonstrated to decrease grain chalkiness, and therefore, improve the quality of visuals and the market value.

CRISPR/Cas systems have also been used to achieve advances in nutritional biofortification. Gene editing of genes that regulate the intake and retention of micronutrients in rice grains, such as *OsNAS, OsZIP,* and *OsVIT,* has led to a higher content of iron and zinc in rice grains. Recent research has paired genome editing with traditional biofortification methods to increase the nutrient levels and bioavailability, which are global deficiencies of micronutrients.^62^ ^,^ ^63^ The invention of base-editing technologies has further enhanced accuracy in engineering the rice genome. The cytosine and adenine base editors have been employed to place specific nucleotide substitutions in grain quality-associated genes without causing any double-strand breakage. For example, base editing has already been used to edit allelic variants of biosynthesis genes of starch, thus allowing precise control of the amylose content and grain texture.^8^ ^,^ ^53^ These methods reduce the number of unwanted mutations and enhance the effectiveness of editing. More recently, prime editing has become a promising technology to enable specific modifications of the genome in rice. Even though it is still in the initial phase of development, research has shown that it can be used to introduce targeted insertions, deletions, and substitutions in key genes. Prime editing is also especially useful in the correction of elite alleles or the introduction of advantageous variants related to the traits of grain quality, which further increases the range of precision breeding.^49^ ^,^ ^64^

Another significant trend is the creation of transgene-free genome-edited rice lines. Researchers have produced edited plants that do not hold foreign DNA by segregating out transgenic elements, or by using ribonucleoprotein (RNP)-based delivery systems. This will help address regulatory issues and increase the willingness of people to accept genome-edited crops.^65^ Even with these developments, there are still challenges, such as off-target effects and low efficiency of precise editing techniques, such as HDR and prime editing, and genotype-dependent transformation efficiency. However, these limitations should be overcome by further enhancements in CRISPR technology, such as engineered Cas variants and AI-assisted design of guide RNA. In summary, current research shows that CRISPR/Cas technologies are irreplaceable in terms of improving the quality of rice grains. The combination of improved editing systems, functional genomics, and breeding measures is accelerating the process of creating a high-quality rice variety that is aligned with the needs and expectations of consumers in terms of quality and nutritional value.

## 10. Applications in biofortification and consumer-preferred rice

Beyond yield improvement, the application of CRISPR/Cas genome editing in rice has been extended to tackling critical problems associated with nutritional security and consumer preference. Improving human health and addressing the market needs are the main breeding goals of biofortification and quality enhancement. Technologies based on CRISPR offer an effective and accurate means to alter genes regulating nutrient accumulation, bioavailability, and sensory properties without introducing foreign DNA.

Micronutrient biofortification, especially of iron (Fe) and zinc (Zn), which are commonly deficient in staple diets, is one of the major applications of CRISPR/Cas in rice. Genome editing of genes such as *OsNAS* (nicotianamine synthase), *OsZIP* (zinc transporters), *OsYSL* (metal transporters), and *OsVIT* (vacuolar iron transporters) has been demonstrated to improve the uptake, translocation, and accumulation of micronutrients in rice grains. For example, nicotianamine production is promoted by the upregulation or targeted modification of *OsNAS,* which facilitates the process of metal chelation and transport, thereby enhancing Fe and Zn loading in the endosperm.^55^ ^,^ ^63^ These approaches are essential in fighting hidden hunger, especially in developing nations. CRISPR/Cas systems have been used not only to increase nutrient content but also to decrease anti-nutritional factors, including phytic acid, which limits the bioavailability of minerals. Refining the genes that are involved in phytic acid production can enhance the bioavailability of the essential micronutrients without adversely affecting plant growth. This two-pronged approach of enhancing the nutrient density of rice and decreasing the number of inhibitors dramatically improves the nutritional value of rice.

CRISPR/Cas technology is also crucial in developing rice varieties that consumers would prefer by targeting characteristics such as aroma, texture, grain appearance, and cooking quality. The introduction of a fragrance in non-aromatic varieties of rice has been widely implemented through editing the *BADH2* gene to enhance the value and acceptance of the produced rice in the market.^43^ Similarly, starch-related genes such as *Wx* and *SSIIa* can be modified to enable the production of rice varieties with customized amylose levels, leading to a higher quality and texture of cooking in the desired manner in different populations. Another important factor that affects consumer preference is grain appearance, including size, shape, and translucency. The editing of rice genes, such as *GS3, GW2,* and *GW5,* using CRISPR/Cas facilitates the production of rice varieties with desirable grain morphology. Also, the editing of *Chalk5* to reduce chalkiness enhances the quality of the visuals and the milling efficiency, further improving the market acceptance.^53^

One of the new fields of use is the production of functional varieties of rice with enhanced health value. The starch composition can be altered using genome editing in order to create rice with a lower glycemic index, which is beneficial in the case of diabetic populations. Likewise, approaches to enhancing protein quality by editing genes involved in the biosynthesis of amino acids, or by modifying metabolism, are both promising and beneficial. Another significant development is the production of transgene-free genome-edited rice lines, which was accomplished by segregation or direct delivery of CRISPR/Cas ribonucleoproteins. These lines do not contain any foreign DNA, making them more acceptable to regulatory bodies and consumers. This technology has been able to bridge the gap between the traditional breeding and transgenic technologies, and provide a sustainable pathway for crop improvement.

Although these developments have occurred, issues such as transformation efficiency that is genotype-dependent, regulatory uncertainties, and potential trade-offs between yield and quality must be tackled. Nevertheless, the current advancements in genome editing technology, along with integrative breeding strategies, are likely to increase the efficiency of CRISPR/Cas in the biofortification and quality improvement of rice. In summary, genome editing using CRISPR/Cas could serve as a potent tool for creating nutritionally enhanced and consumer-desired rice varieties. This technology can play a significant role in ensuring global food and nutritional security, as well as addressing changing consumer needs by targeting important genes that are involved in the metabolism and quality of food and nutrients.

## 11. Limitations and challenges

Although the CRISPR/Cas technologies have tremendous potential to enhance the grain quality traits in rice, several technical, biological, and regulatory barriers restricting their extensive use. These limitations need to be overcome in order to translate genome editing innovations into field-level innovations and commercial success. One of the main issues linked to CRISPR/Cas systems is the presence of off-target effects, in which unintended genomic regions are edited because of partial sequence similarities with the guide RNA. Despite advances in algorithms to design sgRNAs and the creation of high-fidelity Cas variants, it has become difficult to completely eliminate off-target activity. Off-target mutations can result in undesirable phenotypic effects or compromise genomic stability, especially when multiple genes are edited concomitantly.^66^ ^,^ ^67^

The other major restriction is that accuracy editing mechanisms, particularly HDR and prime editing, are not very efficient. In plant systems, such as rice, DNA repair mechanisms largely prefer NHEJ, which is error-prone and does not allow the introduction of specific changes in nucleotide sequences. Although base editing and prime editing technologies have, in part, solved this problem, they are not completely efficient, and many depend on the genotype.^49^ Another challenge is the complicated genetic architecture of the traits of grain quality. The quality of cooking, nutritional content, and appearance of grains are regulated by a complex number of genes and regulatory networks. Modifying only one gene might not yield the desired phenotype, and simultaneous manipulation of multiple genes can increase the chances of an unwanted interaction or pleiotropic effect. For example, any changes in the genes involved in the biosynthesis of starch could cause a change in the quality and yield of grains and lead to trade-offs that should be carefully controlled.

Another key bottleneck to rice genome editing is transformation and regeneration efficiency. Most elite rice varieties and tissue cultures, especially indica types, have been recalcitrant to transformation and tissue culture, limiting the use of CRISPR/Cas technologies. Plant regeneration can also result in somaclonal variation that can confound the impact of desired genome editing. There are also regulatory and biosafety issues that are challenging. Some regulatory systems apply the same rules to transgene-free genome-edited crops, whereas others use more lenient guidelines for transgene-free genome-edited crops. Commercialization policies for genome-edited rice vary considerably across countries. Japan, the United States, and Argentina have adopted relatively flexible regulatory frameworks for certain transgene-free genome-edited crops, whereas the European Union generally regulates genome-edited crops under legislation applicable to genetically modified organisms (GMOs). China has also introduced regulatory guidelines to facilitate the evaluation and commercialization of genome-edited crops while maintaining biosafety oversight. These regulatory differences influence research investment, field testing, commercialization timelines, and international trade, highlighting the need for greater international harmonization of science-based regulatory frameworks. This non-uniformity poses confusion for researchers and breeders and may slow down the commercialization of genome-edited rice varieties.^68^ Moreover, the societal view and tolerance of genome-edited crops are also key determinants of their adoption. Despite the fact that the CRISPR/Cas technology is more accurate than traditional genetic engineering technology, there are still concerns about food safety, environmental effects, and ethical issues. In order to establish trust among the population, effective communication and clear risk assessment are needed. In addition, the harmonization of regulatory policies across countries would facilitate international collaboration, simplify commercialization pathways, and improve the global acceptance of genome-edited rice varieties.

The limitations of the PAM sequences and the variability in the efficiency of editing the system also influence the flexibility of the targeting capability of CRISPR systems. Although engineered versions of Cas have extended the repertoire of editable genomic regions, some loci are challenging to target owing to sequence constraints or chromatin inaccessibility. Finally, the decision to introduce CRISPR/Cas technologies into traditional breeding programs should be properly planned. The edited traits should be stable across environments and generations, and their performance should be confirmed under field conditions. The lack of long-term studies on the stability and ecological impact of genome-edited crops further highlights the need for comprehensive evaluation. To conclude, although CRISPR/Cas-mediated genome editing opens more opportunities to enhance rice, several limitations and challenges that need to be addressed. It is hoped that with the development of genome editing technologies, better transformation methods, regulatory harmonization, and increased public awareness, these barriers will be overcome and that the successful application of CRISPR technologies in rice breeding programs will be adopted.

## 12. Future prospects

The future of CRISPR/Cas-mediated genome editing in rice is set to shift past single-gene manipulations to precision breeding of complex, consumer-centric traits. As soon as the rapid development of genome editing technology is achieved, along with the use of integrative omics and computational tools, the efficiency, accuracy, and applicability of CRISPR systems for improving grain quality are expected to grow significantly. The most promising line is the evolution of genome-edited rice varieties that do not use transgenes, and are more likely to be approved by regulators and accepted by the public. Delivery systems based on ribonucleoprotein (RNP)-based systems, and the use of transient expression methods, allow precise editing without stable integration of foreign DNA. These approaches would make genome editing more consistent with traditional breeding and can be readily commercialized and adopted.

Another new frontier is the integration of artificial intelligence (AI) and machine learning with CRISPR technology. The AI-based tools have the potential to optimize the design of guide RNAs, predict off-target effects, and identify novel gene targets linked to grain quality properties. These methods will serve to increase the efficiency and accuracy of the editing process, especially when dealing with intricate traits that are regulated by a large number of genes and regulatory pathways. Also anticipated in future research is multiplex and pathway-level editing, which allows the simultaneous editing of multiple genes involved in starch biosynthesis, aroma formation, grain morphology, and nutrient accumulation. Such a systems-level approach will enable the creation of rice varieties with combinatorial improvements of traits, including improved cooking qualities, better nutritional value, and desirable grain appearance. The integration of pan-genome resources with genome editing is expected to facilitate the identification of superior natural alleles that can be directly introduced into elite cultivars. This strategy may broaden the genetic diversity available for precision breeding and accelerate the development of superior rice varieties.

Identification of targets and functional validation will be further enhanced with the application of multi-omics approaches, such as genomics, transcriptomics, proteomics, and metabolomics. The combination of these datasets with CRISPR-based editing will allow a better understanding of the relationships between genes and traits and make it possible to design high-quality rice varieties reasonably. Specifically, the analysis of pangenomes can reveal new alleles of various germplasm, which can be new targets of genome editing. Another important field of development is the application of epigenome editing, whereby the CRISPR/dCas systems are used to modify the expression of genes by adjusting DNA methylation or histone modifications without changing the genome sequence. This method provides a reversible and environmentally responsive method for controlling grain quality characteristics, in particular, those whose characteristics depend on environmental factors, such as temperature and water supply.

The idea of climate-resilient quality rice is becoming significant as well. In breeding programs in the future, the focus will be on how to bring together the grain quality specifications and the ability to withstand abiotic stresses such as drought and heat. Cas and CRISPR/Cas technologies could be adopted to simultaneously edit genes related to stress tolerance and quality traits to maintain the same performance under varying climatic conditions. The increase in the speed of breeding and genomic selection is likely to complement CRISPR-based methods, expediting the creation of better rice varieties. A combination of high-rate growth of generations and the precision of genome editing will greatly decrease the number of breeding cycles and increase the gain of genetic information.

Regardless of these opportunities, the effective implementation of CRISPR/Cas technologies will rely on the ability to overcome the existing challenges associated with the efficiency of editing, delivery systems, and regulatory frameworks. The harmonization of international regulations and more widespread public awareness will be instrumental in helping to introduce genome-edited crops. In summary, the future of CRISPR/Cas in the improvement of the quality of rice grains is based on its combination with other advanced genomic tools, computation methods, and sustainable breeding strategies. CRISPR technology has enormous potential to create next-generation rice varieties that can address the twofold objectives of nutritional security and consumer preference in a shifting global environment.

## 13. Conclusion

Overall, genome editing using CRISPR/Cas technology can be considered an innovative strategy that may facilitate the enhancement of several traits that influence grain quality in *Oryza sativa* L., including its culinary properties, taste, appearance, nutritional content, and health benefits. With advancements in genome-editing methodologies, from traditional CRISPR/Cas9-based approaches to next-generation solutions such as base editing, prime editing, and multiplex genome editing, it is feasible to effectively edit genes involved in specific grain quality parameters. In this context, the successful editing of genes controlling starch composition (*Wx*), rice fragrance (*BADH2*), grain elongation (*GS3/GW2*), chalkiness (Chalk5), and the accumulation of nutrients in rice kernels (transporter genes, e.g., *OsNAS, OsZIP, OsYSL,* and OsVIT) has been reported. The application of CRISPR/Cas in functional genomics studies is instrumental for the identification of gene targets that could be further edited to enhance the nutritional content, cooking quality, flavor, or appearance of grains. Notably, contemporary gene editing methodologies make it possible to accurately tune allele variants to induce targeted alterations in the activity or expression levels of genes responsible for grain quality traits without detrimental effects on yields. However, several issues need to be addressed in the future, including off-target mutations, the inefficiency of precise genome editing methods, genotype-dependent constraints for transgenesis, and the regulatory framework for genome editing applications. Overcoming such challenges would involve advancements in gene editing techniques and gene delivery systems. Additionally, considering that grain quality traits involve complex gene networks, their modification might involve a systems biology approach. Finally, the integration of genome editing innovations with emerging technologies might facilitate future progress in grain quality trait modifications in rice. Such developments will enable fast and large-scale development of genome-edited varieties free from foreign genes and multiple gene edits.

## Data Availability

No data was used for the research described in the article.
